# How individuals’ opinions influence society’s resistance to epidemics: an agent-based model approach

**DOI:** 10.1186/s12889-024-18310-6

**Published:** 2024-03-20

**Authors:** Geonsik Yu, Michael Garee, Mario Ventresca, Yuehwern Yih

**Affiliations:** 1 School of Industrial Engineering, Purdue University, Grant St, West Lafayette, 47907 IN USA; 2 Air Force Institute of Technology, Hobson Way, Wright-Patterson AFB, 45433 OH USA; 3 Purdue Institute for Inflammation, Immunology, and Infectious Diseases, Purdue University, Purdue Mall, West Lafayette, 47907 IN USA

**Keywords:** Epidemic modeling, Opinion dynamics, Agent-based simulation, Mass media, News audience polarization, Echo chamber

## Abstract

**Background:**

Protecting public health from infectious diseases often relies on the cooperation of citizens, especially when self-care interventions are the only viable tools for disease mitigation. Accordingly, social aspects related to public opinion have been studied in the context of the recent COVID-19 pandemic. However, a comprehensive understanding of the effects of opinion-related factors on disease spread still requires further exploration.

**Methods:**

We propose an agent-based simulation framework incorporating opinion dynamics within an epidemic model based on the assumption that mass media channels play a leading role in opinion dynamics. The model simulates how opinions about preventive interventions change over time and how these changes affect the cumulative number of cases. We calibrated our simulation model using YouGov survey data and WHO COVID-19 new cases data from 15 different countries. Based on the calibrated models, we examine how different opinion-related factors change the consequences of the epidemic. We track the number of total new infections for analysis.

**Results:**

Our results reveal that the initial level of public opinion on preventive interventions has the greatest impact on the cumulative number of cases. Its normalized permutation importance varies between 69.67% and 96.65% in 15 models. The patterns shown in the partial dependence plots indicate that other factors, such as the usage of the pro-intervention channel and the response time of media channels, can also bring about substantial changes in disease dynamics, but only within specific ranges of the dominant factor.

**Conclusions:**

Our results reveal the importance of public opinion on intervention during the early stage of the pandemic in protecting public health. The findings suggest that persuading the public to take actions they may be hesitant about in the early stages of epidemics is very costly because taking early action is critical for mitigating infectious diseases. Other opinion-related factors can also lead to significant changes in epidemics, depending on the average level of public opinion in the initial stage. These findings underscore the importance of media channels and authorities in delivering accurate information and persuading community members to cooperate with public health policies.

**Supplementary Information:**

The online version contains supplementary material available at 10.1186/s12889-024-18310-6.

## Introduction

Preventative interventions, such as mask-wearing, social distancing, hand-washing, and vaccination, often require active participation from citizens for them to be effective against infectious diseases [27]. Beliefs, opinions, and behaviors have become key elements in disease mitigation during recent pandemics, given the impact of compliance with public health policy. For example, in several countries, participation in these preventive interventions was not sufficiently high to halt the spread of the SARS-CoV-2 virus [13]. Instead, multiple sources disseminated misinformation that fostered distrust in preventive interventions [57], and people exhibited unexpected behaviors [26, 65]. Social aspects of pandemics that are relevant to public opinion, such as polarization and social segregation, have drawn the attention of researchers and were accused of being a threat to public health during the pandemic [32, 34, 35, 40]. Studies by these researchers have shown either the existence of such social phenomenon during the COVID-19 pandemic or the statistical significance of factors on the outcomes of disease spread. However, they have not thoroughly explained the mechanisms of how the consequences of the pandemic could vary under different social conditions, as their approaches are separate from existing epidemic modeling.

The objective of this paper is to examine how varying conditions in opinion dynamics can influence the coevolving disease dynamics and public health through a comprehensive epidemic model. To achieve this, we introduce an agent-based simulation framework that incorporates the change in people’s opinions about interventions over time into an existing disease model, serving as the basis of our study. We calibrate our models using data from 15 countries to demonstrate our framework’s ability to replicate key aspects of the COVID-19 pandemic. These calibrated models also serve as proxies to investigate how different opinion-related factors impact epidemic outcomes. Factors related to opinion dynamics, such as the public’s initial opinion on preventive interventions, media usage for pro-intervention channels, news audience polarization, and echo chambers, are included in our experiments and analysis. We evaluate the permutation importance of each factor in determining the cumulative number of new cases. Additionally, we expand our analysis through partial dependence plots to explore the compounding nonlinear effects of these factors on disease dynamics.

## Related literature

Many sociology, political science, and data science researchers reported that the COVID-19 pandemic is a social problem as it is politicized in the US, UK, and other European countries [32, 34, 35, 40]. Some also claimed that the politicized nature of the pandemic is culpable for public distrust in health-protective interventions [32, 35, 40]. Jiang et al. found that conversations on Twitter regarding COVID-19 in the US are primarily shaped by political affiliation and reveal that there is significant segregation between the two opposing political communities [32]. According to their study, in the US, partisanship is one of the significant factors that drive the evolution of public belief in COVID-19 issues. Kerr et al. also verified the same proposition that the US public responses to the COVID-19 pandemic had been politicized [35]. Jungkunz presented that a similar political polarization in the COVID-19 discussion was found in Germany. Both Kerr et al. and Jungkunz claimed that the COVID-19 pandemic could have been managed more efficiently if we had cross-party consensus on COVID-19 countermeasures [34, 35]. Makridis and Rothwell demonstrated that political affiliation mediates the effectiveness of disease mitigation policies by analyzing Gallup Panel data [40]. The authors also claimed that the adopted policies were moved away from the optimal as a consequence of the politicization of COVID-19. Some other researchers reported observing online “echo chambers” in the context of COVID-19, suspecting that they amplify the spread of misinformation [15, 33, 62], while they could not provide evidence that these echo chambers change the coevolving disease dynamics. The aforementioned studies have emphasized the importance of social aspects in disease mitigation from various perspectives. However, because these studies differ from traditional epidemic modeling approaches, many aspects of COVID-19 remain unanswered, such as changes in disease dynamics under different conditions of opinion dynamics. It is crucial to explore the comprehensive nature of epidemics to develop effective policies that take public opinion into account.

As researchers began to view the COVID-19 pandemic as a social phenomenon rather than just a medical emergency, there were attempts to incorporate social behaviors into epidemic models [10, 37, 47, 51, 63]. She et al. proposed a mathematical model that integrates the disease dynamics of the susceptible-infectious-susceptible (SIS) model with opinion dynamics within a networked population [51]. The authors derived generic results for disease extinction from this combined model through analytical means. Bhowmick and Panja’s study also modeled opinion dynamics and disease dynamics simultaneously on a multiplex network and sought to identify mathematical conditions for disease-free equilibrium [10]. Retzlaff et al. presented an agent-based model that simulates disease spread while considering people’s protective behavior in response to different media messaging, with a focus on how people’s fear grows and diminishes [47]. Wang et al. attempted to find effective policies in the presence of misbehaving agents using a comprehensive model [63]. Kuo and Wen addressed the problem using a data-driven model that incorporated geographical details based on the Taipei metropolitan area [37]. Their model includes parameters related to public awareness and people’s willingness to undergo testing. However, the simplified aspects of the coevolution of public awareness still leave room for further exploration, and there is a lack of consideration for the varied patterns of opinion dynamics observed in different countries during the COVID-19 pandemic.

Despite the efforts and achievements of these studies, the interplay between factors related to public opinion and disease spread still requires further explanation. Specifically, simulation modeling studies that integrate datasets from both opinion dynamics and disease dynamics remain rare due to the high complexity of such models. Also, opinion-related factors in the comprehensive models have received less attention compared to the characteristics of the spreading disease itself despite its importance.

## Model and methods

**Fig. 1:**
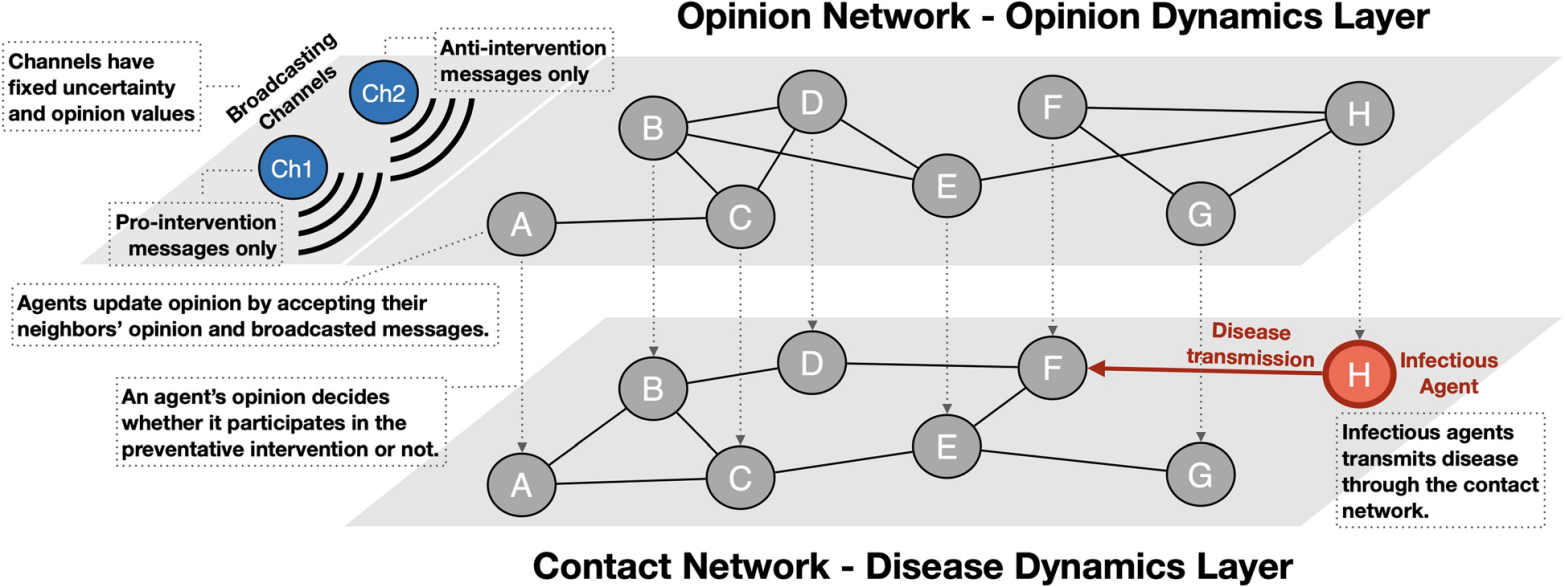
Schematic of the simulation model. Each individual agent (gray or red node) has attribute values that define its behavior and status throughout a simulation run via a given opinion dynamics model. Two channels broadcast their own opinions to the individual agents

We propose an agent-based simulation framework that integrates opinion dynamics and disease dynamics, assuming that an agent’s opinion determines its behavior. As shown in the left-upper corner of Fig. 1, our simulation model is based on the assumption that every agent in the system receives messages from two different mass media channels: each channel broadcasts pro-intervention messages and anti-intervention messages, respectively. The following subsections explain the components and procedures of our agent-based simulation model.

### Network layers

We consider a population of *N* individual agents, and two mass media channels ($\mathcal {M}_1$ and $\mathcal {M}_2$). Our model simulates a polarized mass media environment where two mass media channels broadcast opposing messages regarding preventive interventions. $\mathcal {M}_1$ and $\mathcal {M}_2$ represent these two channels. Agents are connected to each other over two distinct undirected network layers: opinion network ($G_o=(A\cup \{\mathcal {M}_1,\mathcal {M}_2\}, E_o)$) and physical contact network ($G_{c,t}=(A, E_{c,t})$), where *A* denotes the set of vertices that correspond to each individual agent, and $E_{c,t}$ and $E_o$ denote the sets of edges connecting vertices in $G_{c,t}$ and $G_o$, respectively. Agents receive others’ opinions and uncertainty from their neighbors in the opinion network to update their own. Also, agents make physical contact with their neighbors in the contact network and may transmit disease or be exposed to the disease. By neighbors of an agent-*i*, we refer to the set of agents that are connected to the agent-*i* by an edge.

Each network layer is expressed as an undirected, unweighted simple graph with no self-loops, assuming interactions in both networks are mutual. We suppose that the opinion network layer is static, assuming that people share their ideas with their trusted few only. On the other hand, we use different random-generated contact networks with the same degree configuration at each time *t*. This setup enables us to utilize the network-based compartmental model as our approximation model for model calibration. This is because the network-based compartmental model distinguishes the population based on their degrees but does not keep a specific network structure over time. The degree distributions of networks ($G_o$ and $G_{c,t}$) follow Poisson random distributions, which are widely accepted in networked epidemic modeling studies, with mean degrees denoted as $\langle k \rangle _{(o)}$ and $\langle k \rangle _{(c)}$, respectively [9, 59]. Opinion network instances are generated by the social distance configuration (SDC) model suggested by Talaga and Nowak to test the impact of different levels of homophily in the opinion network [55]. Contact network instances are generated by the Newman et al’s configuration model [42].

### Individual agents

Other than its own connections within the two networks, each individual agent has six attribute values that define its behavior and status throughout a simulation run. Some attributes are updated at each time step *t*, whereas other values are fixed. Each agent-*i* has 6 attributes: an opinion value on intervention ($o_{i,t}$), an uncertainty value in their own opinion ($u_{i,t}$), a media usage ratio ($m_i$), a channel usage ratio for $\mathcal {M}_1$ ($g_{i}$), compliance-to-intervention state ($c_{i,t}$), and disease state ($d_{i,t}$).

The opinion value ($o_{i,t}\in [0,1]$) of an agent indicates how strongly an agent agrees to or trusts in the intervention’s effectiveness. $o_{i,t}$ also functions as the probability that the corresponding agent complies with the intervention at time *t*. For example, $o_{13,10}=0.7$ means agent-13 complies with the intervention during the time step $t=10$ with a 70 percent probability. Two types of external influences update an agent’s opinion value. One is from mass media channels, and the other is from one’s neighborhood on the opinion network $G_o$.

The value $u_{i,t}$ describes how uncertain or stubborn an agent is about its own opinion. If an agent’s uncertainty is low, then the agent is stubborn about its opinion and does not change its opinion easily. If an agent’s uncertainty is high, then the agent is likely to change its opinion from external influences. These two attributes follow the definition in the relative agreement (RA) model suggested by Deffuant et al. [17]. As the RA model describes, agents update their uncertainty values whenever they update their opinions. The detailed updating rules are described in the following section as a simulation procedure. We assume that the initial uncertainty is uniform across the general public.

The media usage ratio ($m_i \in [0,1]$) represents an agent’s relative usage of mass media channels (${\mathcal {M}_1, \mathcal {M}_2}$) compared to its neighborhood in the opinion network. In other words, when selecting an information source for opinion updating, the agent chooses either mass media channel $\mathcal {M}_1$ or $\mathcal {M}_2$ with a probability of $m_i$ or its neighborhood in $G_o$ with a probability of $1-m_i$. For problem simplification, the total amount of exposure to new information for individual agents is assumed to be homogeneous.

The channel usage ratio for $\mathcal {M}_1$ ($g_{i}\in [0,1]$) represents the proportion of agent *i*’s $\mathcal {M}_1$ usage out of the agent’s total mass media usage. As we are assuming two different media channels in this study, the channel usage ratio for $\mathcal {M}_2$ is equal to $1-g_{i}$. In the simulation, an agent selects $\mathcal {M}_1$ with probability $g_{i}$ and selects $\mathcal {M}_2$ with probability $(1-g_{i})$ when the agent is in the stage of selecting a mass media channel for opinion attribute updating. We assume that an individual’s media consumption preference represented by these two ratios does not change within the simulated period. For the initialization of $u_{i,t}$, we suppose that all the agents in the system have homogeneous initial uncertainty to simplify our model, following the uncertainty initialization for non-extremist agents in Deffuant et al’s setup [17].

We initialize the three attributes, $o_{i,t}$, $m_{i}$, and $g_{i}$, using a beta distribution with mean and variance values chosen to ensure they fall within the range of 0 to 1, capturing the potential for polarization between two opposing preferences [5]. Beta distributions are well-suited to model bell-shaped and U-shaped patterns, which have been observed in studies of opinion polarization [24, 39, 60]. Specifically for $m_i$ values, we use a bell-shaped distribution, as the preference between mass media and social media is beyond the scope of our research. We set the mean and variance of the distribution to 0.5 and 0.01 for problem simplification, given the complex and unclear nature of people’s preferences in this context [6, 44, 45, 52]. As for the other two attributes, their mean and variance are treated as free parameters for model calibration.

The compliance-to-intervention state ($c_{i,t}$) is a binary attribute. It has a value of 1 when agent *i* complies with the intervention at time step *t* and 0 otherwise. At each time step, each agent sets $c_{i,t}=1$ with probability $o_{i,t}$ and $c_{i,t}=0$ with probability $1-o_{i,t}$.

An agent’s disease state ($d_{i,t}$) shows which state of disease the agent *i* is in. Our simulation model is based on the SEIRS model on networks, which is an agent-based simulation version of the SEIRS model, a common model for diseases with an incubation period and waning immunity [38]. In this disease model, an agent has a disease state, one of “*Susceptible* (*S*)”, “*Exposed* (*E*)”, “*Infectious* (*I*) or “*Recovered* (*R*)”.

### Mass media channel agents

We have two mass media channels ($\mathcal {M}_1$ and $\mathcal {M}_2$) that drive the opinion dynamics. These two channels represent two groups of mass media channels that have a similarity in news content within each group and also have a significant difference between groups. Unlike individual agents, opinion and uncertainty values of $\mathcal {M}_1$ and $\mathcal {M}_2$ are fixed throughout each simulation run, like stubborn agents or extremist agents in the existing studies in opinion dynamics [17, 18, 21, 22, 50]. We fix the opinion values on the intervention that $\mathcal {M}_1$ and $\mathcal {M}_2$ broadcast at 0.9 and 0.1 through a simulation run, respectively, to simulate the media environment with two opposing opinions. That is, $\mathcal {M}_1$’s messages are pro-intervention while $\mathcal {M}_2$’s are anti-intervention.

To simulate varied response times of the media to the pandemic, we initialize the uncertainty values for $\mathcal {M}_1$ and $\mathcal {M}_2$ to be very high (10.0). This ensures that their messages do not impact public opinion or gain serious consideration from the people. After a predetermined response time, we set the uncertainty of the anti-intervention channel at 0.25, while varying the uncertainty of the pro-intervention channel between 0.05 and 0.25 to test different combinations of relative strengths between the two channels. Note that in the RA model, opinions with sufficiently large uncertainty do not influence others’ opinions. These effects combine to create a population of agents that begins to be influenced by mass media channels after a response time has elapsed.

### Simulation procedures

**Fig. 2:**
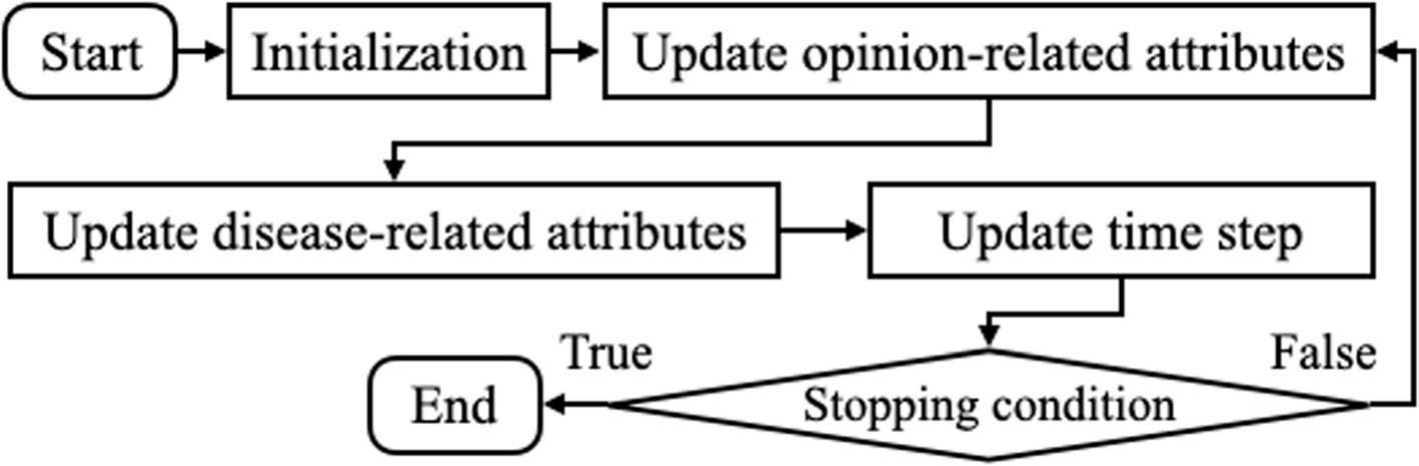
Main ABM simulation procedure for a single trial

Our main simulation consists of two sub-procedures that operate in turn at each time step *t*. In each sub-procedure, all agents are simultaneously activated following the synchronous activation regime [3]. Figure 2 shows the overall sequence of the main ABM simulation procedure for a single trial. After initialization, the sub-procedure of updating opinion-related attributes ($o_{i,t}$ and $u_{i,t}$) is activated first, and then the sub-procedure of updating disease-related attribute ($c_{i,t}$ and $d_{i,t}$) is activated next. The stopping condition is satisfied when the time step (*t*) reaches the designated end time.

The first sub-procedure is based on the RA model proposed by Deffuant et al.. In this sub-procedure, each agent selects an object for interaction from the opinion network, which could be one of the two mass media channels or a randomly selected individual agent from its neighborhood in $G_o$. During this sub-procedure, the attributes $o_{i,t}$ and $u_{i,t}$ of Agent-*i* are updated as follows:

1a $$\begin{aligned} o_{i,t+1}&= o_{i,t} + \mu _o (h/u_\text {ext}-1)(o_\text {ext} - o_{i,t}) \end{aligned}$$

1b $$\begin{aligned} u_{i,t+1}&= u_{i,t} + \mu _u (h/u_\text {ext}-1)(u_\text {ext} - u_{i,t}) \end{aligned}$$

where $\mu _o$ and $\mu _u$ denote the two update rates for opinion and uncertainty, respectively, while $o_\text {ext}$ and $u_\text {ext}$ denote the external opinion and uncertainty chosen for learning. As in the original RA model, updates occur when the condition $h>u_\text {ext}$ is met, where *h* is defined as $h= \min (u_{i,t}, u_\text {ext} + |o_{i,t}-o_\text {ext}|) - \max (-u_{i,t}, |o_{i,t}-o_\text {ext}|-u_\text {ext})$. *h* represents the overlap between the segments of Agent-*i* and the external source. These segments are defined by their opinion and uncertainty, respectively, representing the agents’ ranges of acceptance.

**Fig. 3:**
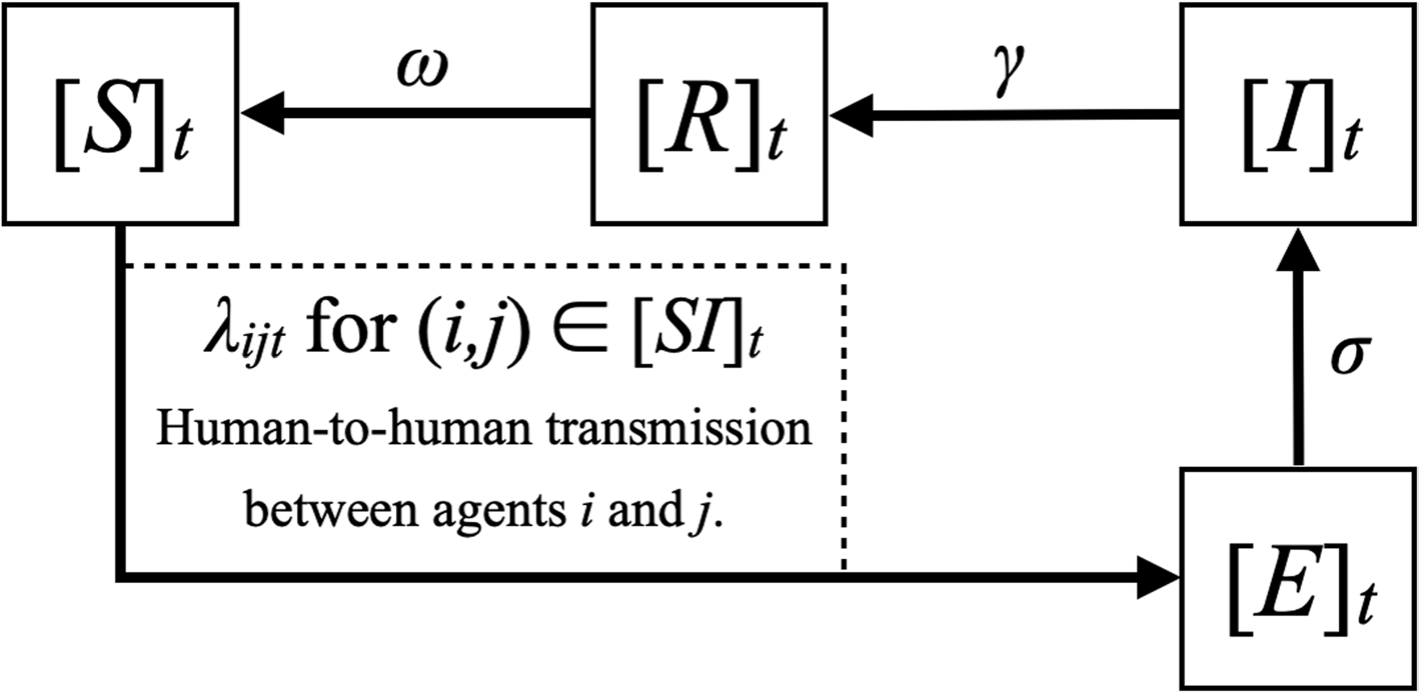
Schematic of the modified SEIRS disease transmission model with variable transmission rate

In the second sub-procedure, we assign 1 to compliance-to-intervention state ($c_{i,t+1}$) with probability of $o_{i,t+1}$ and 0 with probability of $1-o_{i,t+1}$. Then, we determine $d_{i,t+1}$ from $d_{i,t}$ and its neighbors’ disease states at *t*. Figure 3 shows how agents’ disease states change. Each agent in $[S]_t$, $[E]_t$, $[I]_t$, and $[R]_t$ changes its disease state following the arrow with the corresponding probability, where $[A]_t$ denotes the set of agents that are in the disease state of *A* at time *t*. For example, each agent in “*Susceptible*” changes its disease state to “*Exposed*” with probability $\lambda _{ijt}$ for each contact edge ($(i,j)\in [SI]_t$) with its infectious neighbor, where $[SI]_t$ denotes the set of contact edges between susceptible agents ($[S]_t$) and infectious agents ($[I]_t$) at time *t*.

In this study, we assume that the preventative intervention is practically effective in reducing the transmission rate if one of the interacting neighbors cooperates with the intervention (*i.e.* mask-wearing and social distancing). In the simulation, the impact of an agent’s behavior on the intervention is implemented as follows:

2 $$\begin{aligned} \lambda _{ijt} = (1-\rho \max (c_{i,t}, c_{j,t}))\lambda _0 \end{aligned}$$

where $\rho$ denotes the intervention’s effect on the disease transmission rate. For example, masks can block from 67% to 99% of viruses in aerosols, depending on their materials [61]. As reported by multiple studies, there were multiple substantial changes in the intervention policies (e.g., easing of restrictions) and people’s behavior (e.g., summer vacation) during the first summer of the COVID-19 pandemic [12, 23, 67]. To incorporate such temporary changes in a simplified form, we allow our model to adjust the intervention’s effectiveness ($\rho$) during a certain period ($[t_a,t_b]$) by $(1-\delta )$.

### Network-based compartmental model as an approximate counterpart

The network-based compartmental model Eqs. (3)-(9) is used to approximate the disease dynamics of the agent-based model we suggested. The model is modified from the one suggested in Liu and Zhang’s study to use the transmission rate as a function of opinion values [38]. This approximation is used to find the fitted values for disease dynamics parameters more efficiently by avoiding ABM’s heavy computation loads. We first fit opinion parameters using the first submodel of the ABM and then generate the opinion dynamics sequences. Next, we compute the sequence of the average effective transmission rate over all pairs of a random contact network ($\overline{\lambda }_{t}$) with the given mean degree $\langle k\rangle _{(c)}$, so that we can use the sequence as an input to the approximation model.

3 $$\begin{aligned}{} & {} \dfrac{d}{dt}S_k(t) =\omega R_k(t) - \bigg (\dfrac{k\bar{\lambda }_t \cdot \psi (t)}{\langle k\rangle _{(c)}} \bigg ) S_k(t) \end{aligned}$$

4 $$\begin{aligned}{} & {} \dfrac{d}{dt}E_k(t) = \bigg (\dfrac{k\bar{\lambda }_t \cdot \psi (t)}{\langle k\rangle _{(c)}} \bigg )S_k(t) - \sigma E_k(t)\end{aligned}$$

5 $$\begin{aligned}{} & {} \dfrac{d}{dt}I_k(t)=\sigma E_k(t)-\gamma I_k(t)\end{aligned}$$

6 $$\begin{aligned}{} & {} \dfrac{d}{dt}R_k(t) = \gamma I_k(t)-\omega R_k(t)\end{aligned}$$

7 $$\begin{aligned}{} & {} \overline{\lambda }_{t} = \frac{1}{|E_C|}\sum \limits _{e_{ij}\in E_C} \lambda _{ijt}\end{aligned}$$

8 $$\begin{aligned}{} & {} \lambda _{ijt} = (1-\rho (1-\delta \mathbbm {1}_{t\in [t_a,t_b]})\cdot \max (c_{i,t}, c_{j,t})))\lambda _0\end{aligned}$$

9 $$\begin{aligned}{} & {} \psi (t) = \sum _{h}hP(h)I_h(t) \end{aligned}$$

## Parameter fitting and experiment design

The remainder of our study comprises two parts. First, we calibrate our model using two data sequences (i.e., one for opinion dynamics and the other for disease dynamics) from 15 different countries for two primary objectives: demonstrating that our model can replicate specific aspects of the real world and obtaining 15 parameter tuples to build testbeds for examining a selected set of opinion-related factors. Given the widely varied opinion-disease patterns observed across the 15 countries, the 15 testbeds generated through model calibration provide a diverse and viable set of artificial environments for our experiments. Note that Testbeds 1 to 15 are calibrated with datasets corresponding to 15 countries: Australia, Canada, Denmark, France, Germany, Italy, Japan, Netherlands, Norway, Singapore, Spain, Sweden, the UK, the USA, and Vietnam.

In the subsequent sections, we explain the details of the parameter fitting process and provide descriptions of the calibrated parameters. Then we explain the design of experiments that are employed to test the six opinion-related factors. These experiments are conducted based on each of the testbeds 1 to 15.

### Data and fitting methods

We calibrated the submodel of opinion dynamics using data from the YouGov survey [20]. We utilized responses to the question, ‘How often have you worn a face mask outside your home (e.g., when using public transport, visiting a supermarket, or going to a main road)?’ as proxies for people’s opinions on preventive interventions. We quantified the answers by assigning values of 0.0, 0.25, 0.5, 0.75, and 1.0 to the responses ‘Not at all,’ ‘Rarely,’ ‘Sometimes,’ ‘Frequently,’ and ‘Always,’ respectively, assuming the responses were evenly distributed within the interval [0, 1]. Next, we calculated weighted averages of these values within each 5-day time frame. To account for time frames with no data points, we performed linear interpolation using the nearest available data points. The submodel of disease dynamics is calibrated using WHO data on COVID-19 new cases [66]. We use both datasets, specifically the observations from the first 250 days starting from April 1, 2020, which predates the introduction of COVID-19 vaccination. This is because the model only incorporates non-pharmaceutical interventions. We also assume that 48% of the total cases are reported based on Schulman et al’s report [49] and adjust the WHO data, accordingly.

We have fitted 10 opinion model parameters ($O_1$,...,$O_{10}$) and 7 disease model parameters to data from 15 countries. The ranges and granularity of these parameters are shown in Tables 1 and 2. Because our model assumes no feedback loop from the disease model to the opinion model, we fit the two sets of parameters, separately: opinion model parameters first to the data derived from the YouGov survey, and disease model parameters later to the WHO daily new cases data. This reduces the complexity of the parameter space exploration to find the fitted tuples. We also use the approximation model Eqs. (3)-(9) instead of the agent-based model in disease-related parameter fitting to reduce the computational burden. The similarity scores measured in RMSE between the approximation models’ results and the agent-based models’ results are attached in the Appendix.

For the model fitting method, we utilize basin-hopping with multiple starting points (200 in each stage, generated by using Latin hypercube sampling with minimax correlation criterion) as a global search optimizer, and sequential quadratic programming as a local search optimizer [36, 43]. The objective of the fitting process is to minimize the root mean square error (RMSE) between the data and the simulated sequence in each step.

**Fig. 4:**
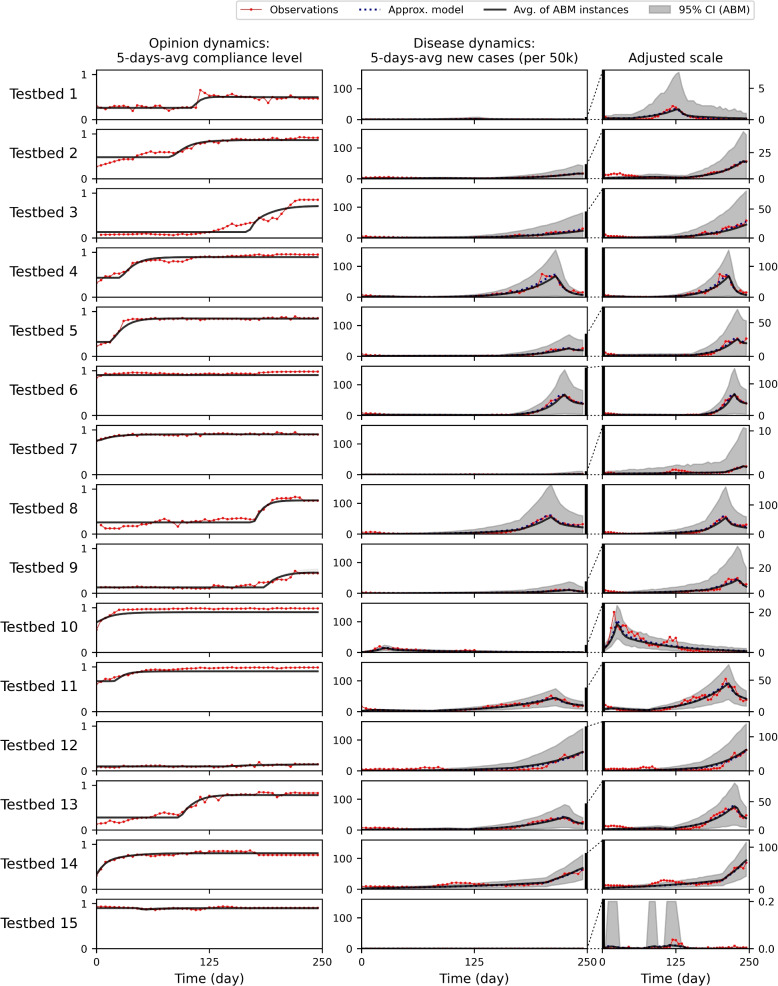
Observed 5-day average opinion (average compliance level to intervention, 1st column) and disease (average new cases, 2nd and 3rd columns) sequences and their corresponding simulated sequences of an agent-based model for 15 countries. Plots in the 3rd column have adjusted y-axis scales to present the details

In Fig. 4, we show the results of parameter fitting for data sets from 15 countries (Testbeds 1 to 15). Opinion dynamics are shown in the first column and disease dynamics are shown in the second and third. Data sequences (YouGov data and WHO data) are presented in red lines, while the average sequences of 2,000 simulated ABM instances are plotted in black lines with 95% confidence intervals in grey areas. The results from the approximation disease model are plotted in blue dotted lines. The results show that the combination of opinion and disease models of our choice can reproduce aspects of the selected datasets. We explain the calibrated parameter values of each testbed and their fitting scores in the Appendix.

Note that we chose a population size of 50,000 to ensure that new cases in each time step exceed 1 in most scenarios. Agent-based models cannot represent fractional numbers of individuals in disease statuses, unlike compartmental models. This limitation prevents us from replicating disease sequences where most numbers are smaller than 1.0. As a result, our model does not yield stable results for the testbed based on datasets from Vietnam. Due to computational resource constraints, we opted for a population size of 50,000, necessitating the exclusion of the case. As shown in the last row of Fig. 4, the new cases per 50,000 are less than 1, resulting in a 95% confidence interval that is inconsistent with the average sequence.

### Opinion-related parameters

This subsection lists and explains the opinion model parameters. Table 1 provides detailed information about these parameters ($O_1, \dots , O_{10}$).

**Table 1:** The parameter space of opinion dynamics submodel

Parameter Description	Notation	Range	Granularity
Mean of the initial opinions on intervention^1^	$O_1$	[0.10, 0.90]	0.010
Variance of the initial opinions on intervention^1^	$O_2$	[0.01, 0.08]	0.010
Mean of the usage for the pro-intervention channel^1^	$O_3$	[0.50, 0.85]	0.010
Variance of the usage for the pro-intervention channel^1^	$O_4$	[0.01, 0.12]	0.010
Level of homophily in the opinion network^2^	$O_5$	[1.0, 8.0]	0.500
Response time of channels to disease spread	$O_6$	[0, 200]	1.000
Uncertainty of the pro-intervention channel’s message^3^	$O_7$	[0.05, 0.25]	0.001
Individual agents’ initial uncertainty^3^	$O_8$	[0.50, 1.50]	0.001
Convergence rate: individual’s opinion^3^	$O_9$	[0.15, 0.25]	0.001
Convergence rate: individual’s uncertainty^3^	$O_{10}$	[0.15, 0.25]	0.001

Remarks: ^1^Beta distribution; ^2^SDA model; ^3^RA model [17]

**Mean (** $\varvec{O_1}$ **) and variance (** $\varvec{O_2}$ **) of the initial opinion values.** $O_1$ represents the mean of people’s initial level of agreement with the authority’s intervention. $O_2$ is the variance of opinion values at $t=0$.

As we can observe in the YouGov data sequences in Fig. 4, every population reacts to non-pharmaceutical interventions differently when it is initially introduced to them during the early stage of COVID-19 [20]. The initial opinion values of agents on intervention ($\{o_{i,0}|i=1,\dots , N\}$) represent this difference in people’s first impressions of non-pharmaceutical interventions (NPIs). As mentioned in “Individual agents” section, we use a beta distribution defined on the interval [0, 1] for $o_{i,t}$ generation while manipulating the two shape parameters to achieve the mean and variance of $O_1$ and $O_2$.

**Mean (** $\varvec{O_3}$ **) and variance (** $\varvec{O_4}$ **) of pro-intervention news channel usage.** Our model has two channels with opposing attitudes toward preventative intervention. $O_3$ and $O_4$ describe the distribution of people’s usage of the pro-intervention news channel ($g_i$). $O_3$ represents the society’s average usage of the pro-intervention channel. $O_4$ describes how much peoples’ usage values deviate from the mean ($O_3$).

Our model is designed to implement Prior and Webster’s concept of polarization, which refers to the tendency of audiences to polarize around two distinctive classes of content [46, 64]. This is because polarization regarding news consumption is considered one of the social aspects that aggravated the COVID-19 pandemic [35]. The polarization can be manipulated by $O_4$ in our model, as high $O_4$ means that there are more people with extreme channel preferences. We sample values from a beta distribution defined on the interval [0, 1] to generate $g_{i}$’s, manipulating the two shape parameters to achieve the mean and variance of $O_3$ and $O_4$. We use beta distribution to mimic the description of polarized distributions in Prior’s study [46].

**Level of homophily in the opinion network (** $\varvec{O_5}$ **)** is the level of homophily parameter used in the SDC network generator suggested by Talaga and Nowak [55]. Note that we compute social distances between agents based on their static characteristics value pairs $(g_i, m_i)$. The homophilic structure in opinion-sharing networks, often referred to as “echo chambers,” is a major concern during COVID-19 [15, 33, 62]. It is suspected to amplify the spread of misinformation, thus hindering disease mitigation efforts.

**Response time of channels to disease spread (** $\varvec{O_6}$ **)**. There exists at least one significant change in each of the 15 sequences of compliance level data. We assume that this change is driven by channels’ starting to broadcast certain messages about NPIs. In our model, $O_6$ is the number of time steps that two mass media channels wait until they start broadcasting messages about NPIs with low enough uncertainty.

The remaining parameters of the relative agreement model ($\varvec{O_7},\dots ,\varvec{O_{10}}$) are also calibrated in the model fitting. Uncertainty of the pro-intervention channel’s message ($O_7$) represents the strength of persuasion of the pro-intervention channel that is explained in “Mass media channel agents” section (i.e., the uncertainty of $\mathcal {M}_1$). Individual agents’ initial uncertainty ($O_8$) describes how susceptible the people are to the external information at the beginning ($\{u_{i,0}|i=1,\dots , N\}$). The two convergence rates of opinion and uncertainty ($O_9$ and $O_{10}$) represent how fast the corresponding attributes converge in the simulation and are used in the updating rules (Eqs. 1a and 1b).

### Disease-related parameters

The proxy values reported in existing COVID-19 studies are used for the following three parameters: $1/\sigma$ (expected duration of the incubation period, 6.5 days), $1/\gamma$ (expected duration of the infectious period, 18 days), and $1/\omega$ (expected duration of the natural immunization, 180 days) [2, 14, 16]. In the fitting stage of the disease submodels, we assume that the three characteristics of the infectious disease are equivalent in all 15 models. We calibrate the following seven parameters to fit the approximation model to the data sequences by minimizing the RMSE between the simulated sequence and the new cases data: transmission rate per contact ($\lambda _0$), the average degree of contact networks ($\langle k \rangle _{(c)}$), the effectiveness of NPI compliance ($\rho$), size of the infectious population at $t=0$ ($|[I]_0|$), starting time of the temporary change in $\rho$ ($t_a$), stopping time of the temporary change in $\rho$ ($t_b$), and rate of the temporary change in $\rho$ ($\delta$). Table 2 shows the descriptions, notation, and ranges of disease-related parameters. The UB in the table is the total number of new infections that occurred during the three-month period in each of the country’s data until April 1st, 2023. We also set the range of the $\delta$ differently for Testbed 10, built using the Singapore case data, $(-0.5, 0.0)$, while not allowing $\delta$ to lead to a negative $\lambda$. No optimal parameter tuple that mimics Singapore’s disease dynamics data is found within the original parameter range. This is because Singapore’s case has a sharp peak in the very early stage and near eradication of the disease in the later stage, which is different from the other cases, which tend to have greater peaks during or after summer.

**Table 2:** The parameter space of disease dynamics submodel

Parameter Description	Notation	Range	Granularity
Transmission rate per contact^1^	$\lambda _0$	(0.014, 0.041)	Rational
Average degree of contact networks^2^	$\langle k \rangle _{(c)}$	(6.0, 15.0)	0.1
Effectiveness of NPI compliance^3^	$\rho$	(0.67, 0.9999)	Rational
Size of the infectious population at $t=0$ (per 50,000)	$|[I]_0|$	(5, UB)	Integeral
Starting time of the temporary change in $\rho$	$t_a$	(0, 210)	Integeral
End time of the temporary change in $\rho$	$t_b$	($t_a, 250$)	Integeral
Degree of the temporary change in $\rho$	$\delta$	(0.0, 0.9)	Rational

Remarks: ^1^[58]; ^2^[8, 31, 48, 54, 69]; ^3^[61]

### Experimental design

To report the six opinion-related factors’ ($O_1, \cdots , O_6$) impact on the disease spread, we generate 10,000 distinct tuples from the ranges shown in Table 1 using Latin hypercube sampling with the minimax correlation criterion. Each of the 10,000 tuples of parameters is examined with the 15 models fitted to different countries’ data. That is, all other parameter values except for the 6 factors of interest are fixed to the ones used in Fig. 4. For further analysis, we track the number of total new infections during the simulation time ($Y_C$) and use the log base 10 of the values as the response variable. The log transformation is applied because epidemics tend to grow exponentially under unfavorable conditions, rather than linearly in size.

## Results

**Fig. 5:**
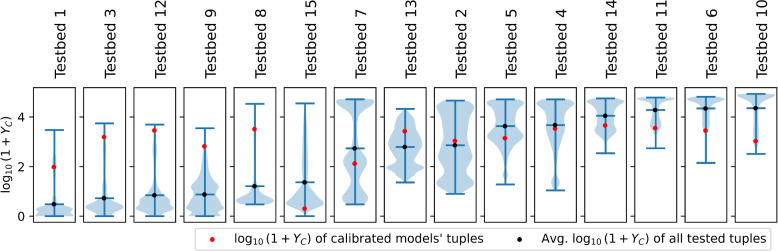
Violin plots of the log-scaled epidemic size ($Y_C$) distributions of 10,000 simulation runs based on each of 15 testbeds. Each simulation run is based on a distinct opinion-related parameter tuple ($O_1,\dots ,O_6$). Plots are sorted by the mean of the log-scaled epidemic size. Epidemic sizes are measured in the number of total new infections during the simulation time in a virtual social system with a 50,000 population size. The red dots in the figure represent the response values ($Y_C$) of the models fitted to the corresponding data. Plots show that the consequence could be much better or much worse depending on the given conditions of opinion dynamics

In this section, we analyze the outcomes of 10,000 different tuples of the six opinion-related factors for each of the 15 testbeds. We use random forest regression for result analysis, which is an ensemble learning method based on a collection of decision trees [11]. This choice is motivated by its capability to yield robust models with relatively high $R^2$ values, even when dealing with nonlinear and complex relationships between factors and the response [4]. The distributions of the 10,000 simulation outcomes based on each of the 15 testbeds are displayed as log-scaled violin plots in Fig. 5. The shapes and locations of these distributions are determined by the 11 parameters that are fixed and not tested in this section. A lower mean value indicates that the fixed parameter set tends to result in relatively small numbers of cumulative new cases, and vice versa. For instance, a small mean degree in the contact network can lead to decreased $Y_C$, in general.

Note that in this paper, our focus is on presenting results for the first six opinion-related parameters ($O_1,\dots ,O_6$). This selection arises from the relatively greater difficulty in identifying counterparts indicating the remaining four parameters ($O_7,\dots ,O_{10}$) in the YouGov survey or in other sources. This difficulty also leads to retrieving clear implications from the analysis result. The extended results, including all 10 opinion-related parameters, can be found in Appendix B, where the results of the factor importance analysis and partial dependence plots are provided for all 10 parameters.

In the following subsections, we measure the normalized permutation importance of each of the six opinion-related parameters using the random forest models and their corresponding $R^2$ values. Permutation importance, an analysis technique commonly used with ML-based models such as ones generated by random forest regression, quantifies the decrease in the model score resulting from the random shuffling of factor values [11]. For our results, we compute the average score loss over 30 random shuffles. Next, we explore the partial dependencies between the response and factors to understand the marginal effect of factors on the outcome of our explanatory model. Partial dependence plots illustrate the dependence between the response and a set of input parameters of interest, marginalizing the values of all other parameters [28]. As a model-agnostic interpretation method for revealing how the model behaves as a result of changing inputs, it is broadly accepted in various fields, including medical studies and disease modeling [30, 41, 53].

### Factor importance analysis: dominance of $O_1$

Table 3 reports the normalized permutation importance of the six opinion-related factors in 15 different models. The mean of the initial opinions on intervention ($O_1$) dominantly affects disease spread in all 15 models, with importance values ranging between 0.6967 and 0.9665. In most cases, the importance of $O_6$ has the second-largest value, ranging between 0.0118 and 0.1912. The remaining factors appear to have a limited impact in comparison to $O_1$ and $O_6$ in response.

**Table 3:** Normalized permutation importance (sum to 1.0) of 6 opinion-related factors in their corresponding random forest regression models and their $R^2$ values. The 15 explanatory models are generated based on the results of 10,000 simulation instances, each with the corresponding testbed. The result shows that, among the 6 factors, the average of the public’s initial opinion values ($O_1$) dominates the rest in terms of the permutation importance

Basis	$R^2$	Permutation Importance of Opinion Model Factors					
		$O_1$	$O_2$	$O_3$	$O_4$	$O_5$	$O_6$
Testbed 1	0.8082	0.8453	0.0622	0.0232	0.0107	0.0091	0.0496
Testbed 2	0.8491	0.8477	0.0024	0.0485	0.0027	0.0007	0.0980
Testbed 3	0.8490	0.7972	0.0304	0.0307	0.0137	0.0070	0.1209
Testbed 4	0.7969	0.8970	0.0024	0.0308	0.0034	0.0014	0.0650
Testbed 5	0.8831	0.8699	0.0030	0.0495	0.0043	0.0007	0.0726
Testbed 6	0.9573	0.9046	0.0006	0.0312	0.0100	0.0002	0.0535
Testbed 7	0.8233	0.9351	0.0406	0.0067	0.0043	0.0016	0.0118
Testbed 8	0.7931	0.7609	0.0230	0.0198	0.0029	0.0023	0.1912
Testbed 9	0.8720	0.7834	0.0338	0.0595	0.0289	0.0081	0.0863
Testbed 10	0.9898	0.9429	0.0087	0.0028	0.0010	0.0001	0.0445
Testbed 11	0.9786	0.9665	0.0029	0.0085	0.0011	0.0002	0.0207
Testbed 12	0.8548	0.8751	0.0761	0.0095	0.0086	0.0074	0.0233
Testbed 13	0.8483	0.8215	0.0047	0.0277	0.0061	0.0022	0.1378
Testbed 14	0.9670	0.8581	0.0003	0.0535	0.0056	0.0003	0.0821
Testbed 15	0.7698	0.6967	0.0285	0.1091	0.0350	0.0143	0.1163

The high scores of $O_1$ and $O_6$ in the permutation importance remind us of the importance of early reaction in the effectiveness of disease mitigation [25, 29]. Even though the public’s opinion can be changed over time, it is crucial to have people’s support in disease mitigation as early as possible. The result also implies that, for future epidemic situations, it is necessary to strengthen or maintain the social norm of wearing masks, social distancing, and general trust in scientifically proven interventions [1, 7]. Our result shows that maintaining high $O_1$ can function as a behavioral vaccination to the community for any future pandemic.

### Further analysis with partial dependencies

**Fig. 6:**
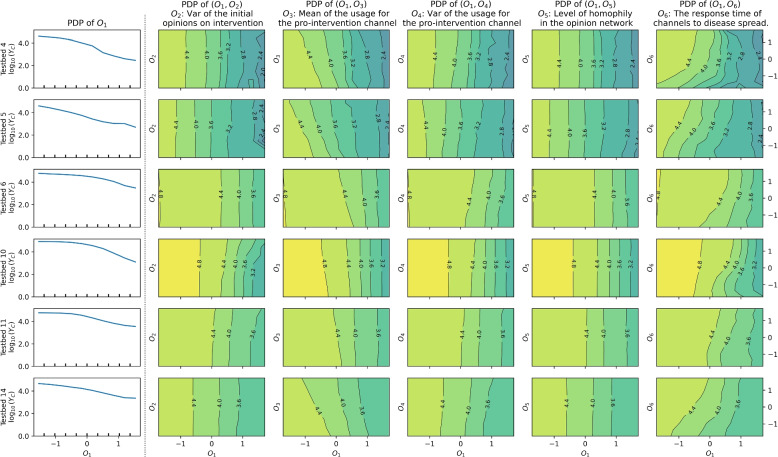
One one-way ($O_1$) and five two-way partial dependence plots of the regression model for the cases in Group 1: Results based on Testbeds 4, 5, 6, 10, 11, and 14

**Fig. 7:**
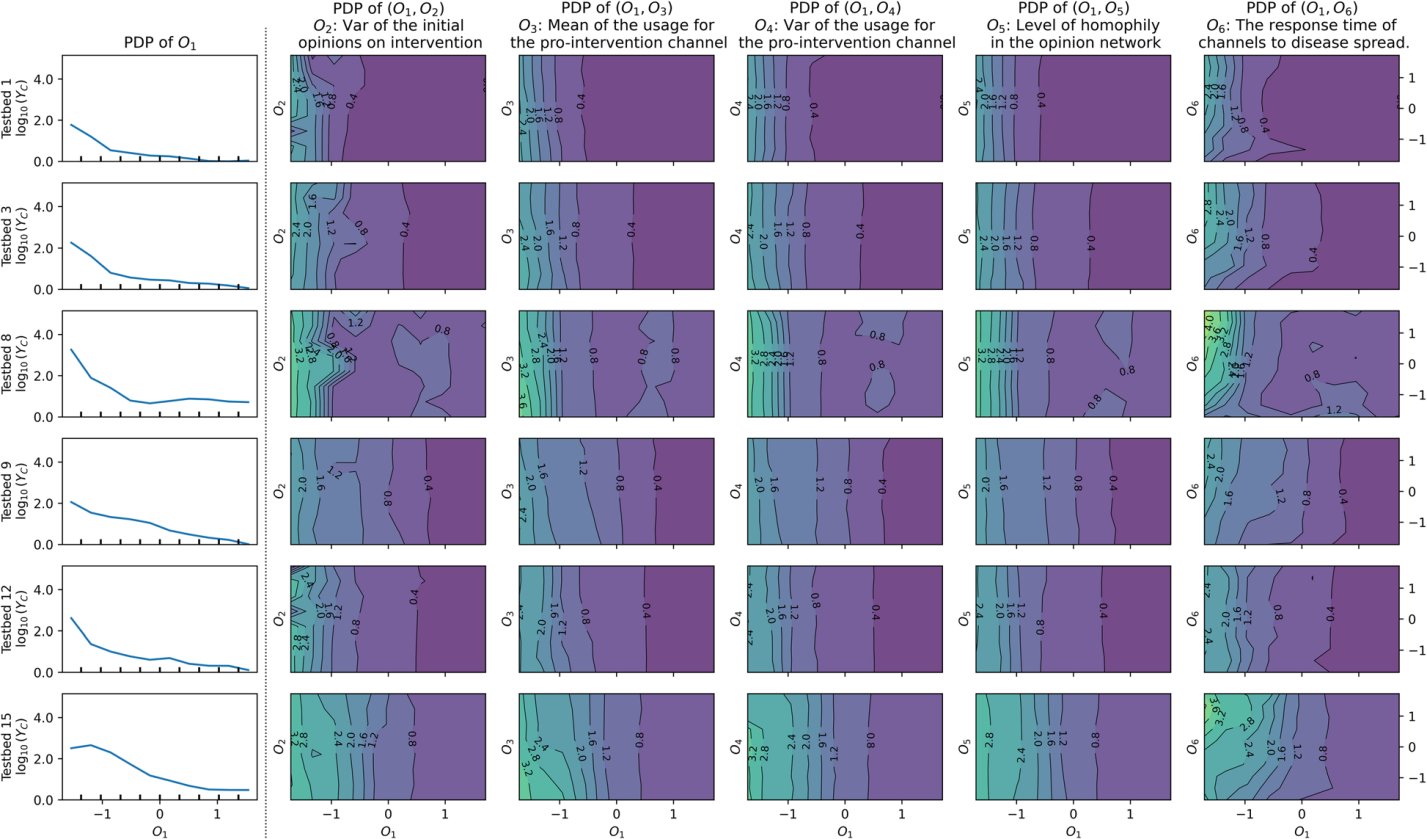
One one-way ($O_1$) and five two-way partial dependence plots of the regression model for the cases in Group 2: Results based on Testbeds 1, 3, 8, 9, 12, and 15

**Fig. 8:**
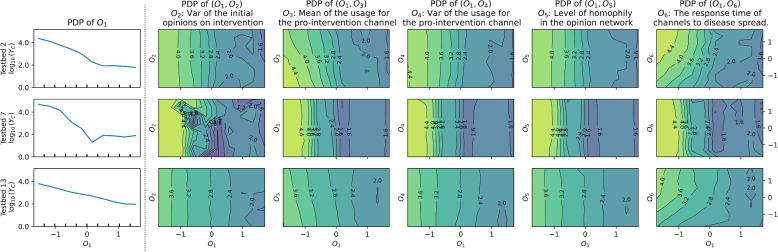
One one-way ($O_1$) and five two-way partial dependence plots of the regression model for the cases in Group 3: Results based on Testbeds 2, 7, and 13

This section explores partial dependence plots of factors in 15 different scenarios and explains the marginal effects of individual factors or pairs of factors on disease spread outcomes. We have clustered the 15 cases into three groups using the K-means algorithm on partial dependence vectors. Each of Figs. 6, 7 and 8 consists of partial dependence plots of models in one of the three groups, respectively. Since the mean of initial opinions on intervention ($O_1$) has a dominant impact on the response variable, one-way partial dependence plots of $O_1$ are presented in the first columns. Two-way partial dependence plots for ($O_1$, $O_j$) pairs with $j\in {2,\dots ,6}$ in Figs. 6, 7 and 8 are shown in following columns and explained below.

Plots of the first columns of Figs. 6, 7 and 8 present the impact of $O_1$, which represents the mean of initial opinions regarding intervention. The downward right patterns in the plots show that a higher value of $O_1$, or citizens’ cooperation in preventive intervention at $t=0$, is advantageous in disease mitigation. Also, contour lines are vertical or have steep slopes in all the two-way partial dependence plots, indicating that the change in epidemic size is driven by $O_1$ compared to the other factors in each plot.

The variance of initial opinions on intervention ($O_2$ in the second columns of Figs. 6, 7 and 8) has a negligible effect on total new infection ($Y_C$), except for the results baes on Testbeds 10 and 11. For the two cases, contour lines are bending to the right, indicating that a higher variance ($O_2$) leads to increased $Y_C$ when $O_1$ is high. Higher variance ($O_2$) under the same average ($O_1$) indicates a greater presence of individuals with extreme opinions on both the anti-intervention and pro-intervention sides. The pattern in both models suggests that having more people who initially detest the preventive interventions can undermine disease mitigation efforts and the increased number of people who advocate the intervention cannot offset the effect.

Next, the mean of the usage for the pro-intervention channel ($O_3$) shows contour lines bent to the left. This pattern suggests that having more news audience for the pro-intervention channel (or high $O_3$) is advantageous to disease mitigation. The curves are rather vertical given high $O_1$, indicating that the importance of $O_3$ diminishes if a pro-intervention public consensus exists. In a real-world scenario, securing more channels to advocate for pro-intervention measures would resemble the conditions associated with a high value of $O_3$.

The variance of the usage for the pro-intervention channel ($O_4$), related to news audience polarization, exhibits right-bent patterns in Group 1 models when $O_1 > 0.5$, while showing vertical contour lines otherwise. In Group 1 cases, a high $O_4$ tends to increase $Y_C$ when $O_1$ is high in the corresponding partial dependence plots. That is, higher news audience polarization can lead to a greater number of total infections, as warned by existing studies [32, 35, 40], but only when $O_1$ is sufficiently high.

The opinion network’s homophily level ($O_5$) primarily results in vertical contour lines, indicating that it has a marginal effect on disease spread in our model where media channels drive opinion dynamics.

The response time of media channels to disease spread ($O_6$) displays clear right-bent patterns in the contour lines of Groups 1 and 3. This shows that media channels’ early reaction to disease spread (characterized by low $O_6$) decreases the total damage caused by epidemics, in general. For models in Group 2, this pattern is less evident. The fitted parameters of Group 2 models commonly show a weak pro-intervention message with high uncertainty, along with either low average opinion over time (Testbeds 1, 3, 8, 9, and 12) or early convergence (15). These characteristics weaken the impact of pro-intervention messages, consequently diminishing the significance of media channels’ early response to disease.

## Conclusion

During the recent SARS-CoV-2 pandemic, understanding the dynamics of public opinion emerged as a key aspect of safeguarding public health. This study aims to shed light on aspects of the pandemic related to public opinion through an agent-based simulation framework. We developed an agent-based model that integrates disease spread and opinion dynamics, based on the assumption that all mass media can be represented as two opposing channels influencing opinion dynamics. Using this model, we replicated the coevolution of opinion and disease dynamics observed during the COVID-19 pandemic in 15 different countries and demonstrated the importance of opinion-related factors in public health and disease mitigation. Our work, therefore, underscores the practical utility and necessity of tracking public opinion and social behaviors to prepare for future disease spread. Our approach also incorporates the aspects of opinion dynamics, such as polarization in media consumption and homophilic network structures in opinion sharing. Despite the accumulating academic attention to social phenomena such as polarized media consumption and opinion clusters with misinformation, academic trials to explain how much they accelerated the disease spread were relatively scares. Our attempt adds to another possibility of quantifying the contribution of concerned social phenomena to the spread of disease.

Our result shows that the mean of people’s opinions in the initial stage outweighs all other factors. This finding aligns with the significance of early action and preparedness for disease spread, as emphasized in multiple existing research articles [25, 29], indicating that epidemics are easier to mitigate when the infectious population is still relatively small. The importance of public consensus on how to respond to epidemics also implies the need to strengthen trust in health recommendations based on science. Achieving this goal may require general health education regarding potential future epidemics, as demonstrated in previous epidemics such as acquired immune deficiency syndrome (AIDS) [19]. Building on our results, we offer additional advice for health authorities to maintain a consistent level of health education about infectious diseases for the general public. This is particularly crucial in the current context, where people still remember the damage caused by COVID-19 and recognize the importance of collective efforts, such as complying with non-pharmaceutical interventions, in mitigating the disease. Maintaining the social norm of adhering to scientific health recommendations through education should be considered a crucial aspect of infectious disease preparedness and thus needs authorities’ consistent attention and investment.

Our study also reveals that other opinion-related factors can have a significant impact on epidemics depending on the mean of public opinions in the initial stage. The results suggest that increasing the audience for channels supporting pro-intervention messages, reducing news audience polarization, and ensuring immediate media responses to potential diseases could significantly help mitigate disease spread. These findings emphasize the need for media channels and information-related authorities to deliver accurate information and persuade community members to cooperate with their policies [56]. Attempts to politicize or polarize public health and epidemic issues may undermine our efforts to mitigate the spread of diseases.

The insights from our study are based on multiple problem simplifications and assumptions, inviting ongoing discussion and further research. Our model relies on the RA framework and specific assumptions about media dynamics. Furthermore, our results require additional country-specific details to be employed as a predictive model for each country. Additionally, while we assume independence among the tested opinion-related factors, real-world correlations may differ. Our model does not distinguish between different levels of disease severity and does not include death [68]. We expect that incorporating these details into the model using data will yield more meaningful results for policymakers in our future study.

Designing and deploying effective opinion interventions (e.g., advertisements and campaigns) during an ongoing pandemic is a complex problem, particularly when considering the challenges of countering the spread of misinformation and fear. In our future work, we aim to suggest efficient opinion intervention policies for disease control by incorporating deeper understandings of people’s behavior from the perspectives of social psychology and media studies.

### Supplementary Information

**Supplementary Material 1.**

## Data Availability

The datasets used and/or analyzed during the current study are available from the corresponding author upon reasonable request.
