# Supplementary material for: How individuals’ opinions influence society’s resistance to epidemics: an agent-based model approach
Source: BMC Public Health. 2024 Mar 20;24:863. doi: 10.1186/s12889-024-18310-6 (PMC10953238; doi:10.1186/s12889-024-18310-6)
Supplement: Supplementary file 1 — Supplementary Material 1. [file 12889_2024_18310_MOESM1_ESM.pdf]

Appendix A. Fitted Parameter Tables

Table S1

Parameter tuples used to reproduce opinion dynamics (compliance level) observed in 15 countries. The root mean squared error (RMSE) is computed between the average sequence of 2,000 ABM instances with a population of 50,000 and data. Note that Testbeds 1 to 15 are calibrated with datasets corresponding to 15 countries, namely Australia, Canada, Denmark, France, Germany, Italy, Japan, Netherlands, Norway, Singapore, Spain, Sweden, the UK, the USA, and Vietnam, in that order.

Basis	Fitted value of opinion-related parameters										RMSE
	O_1	O_2	O_3	O_4	O_5	O_6	O_7	O_8	O_9	O_{10}	
Testbed 1	0.26	0.06	0.61	0.09	7.0	109	0.064	0.78	0.246	0.197	0.0521
Testbed 2	0.48	0.04	0.67	0.01	1.0	84	0.059	1.02	0.171	0.209	0.0716
Testbed 3	0.13	0.07	0.85	0.02	1.5	171	0.205	0.97	0.156	0.173	0.0968
Testbed 4	0.43	0.05	0.85	0.01	2.5	29	0.128	1.04	0.165	0.168	0.0541
Testbed 5	0.32	0.05	0.83	0.01	1.5	19	0.108	0.82	0.183	0.172	0.0353
Testbed 6	0.90	0.02	0.65	0.08	8.0	126	0.100	0.77	0.237	0.217	0.0480
Testbed 7	0.76	0.01	0.64	0.01	3.5	5	0.250	0.52	0.188	0.194	0.0174
Testbed 8	0.26	0.03	0.79	0.10	4.0	177	0.128	1.17	0.213	0.205	0.0674
Testbed 9	0.13	0.02	0.84	0.03	2.0	189	0.196	0.86	0.189	0.204	0.0388
Testbed 10	0.67	0.01	0.76	0.02	6.0	3	0.056	0.58	0.162	0.238	0.0810
Testbed 11	0.68	0.02	0.85	0.05	8.0	23	0.185	0.54	0.233	0.234	0.0666
Testbed 12	0.10	0.06	0.74	0.06	6.5	147	0.141	0.66	0.190	0.238	0.0171
Testbed 13	0.28	0.07	0.73	0.01	6.0	95	0.110	0.96	0.185	0.180	0.0637
Testbed 14	0.29	0.01	0.85	0.05	3.5	1	0.072	1.11	0.151	0.210	0.0352
Testbed 15	0.90	0.03	0.55	0.06	1.5	49	0.070	0.87	0.215	0.242	0.0165

Table S2

This table provides the parameter tuples used to replicate the observed dynamics of new COVID-19 infections in 15 countries based on opinion trends generated using the corresponding tuples from Table 1. We calculate two root mean squared errors (RMSE) to assess the model's performance. RMSE 1 compares the average sequence of 2,000 agent-based model (ABM) instances with a population of 50,000 to the new infection data. RMSE 2, on the other hand, compares the average sequence of 500 ABM instances to the sequence generated by the corresponding approximation model.

Basis	Fitted value of disease-related parameters							RMSE 1	RMSE 2
	λ_0	$\langle k \rangle_{(c)}$	ρ	$\lvert [I]_0 \rvert$	t_a	t_b	δ		
Testbed 1	0.0142	6.0	0.9999	6	53	131	0.9000	0.2899	0.1050
Testbed 2	0.0210	6.5	0.7407	15	146	244	0.4630	1.5702	0.3525
Testbed 3	0.0140	6.0	0.9865	7	122	250	0.8810	2.5577	1.5898
Testbed 4	0.0209	7.2	0.9356	47	82	218	0.6407	6.1025	3.3036
Testbed 5	0.0141	10.8	0.6742	13	155	231	0.5071	2.3927	1.0156
Testbed 6	0.0176	13.4	0.8076	73	167	226	0.5460	3.6230	2.5081
Testbed 7	0.0140	12.0	0.6700	5	211	242	0.3928	0.2475	0.0287
Testbed 8	0.0173	7.0	0.7438	5	178	212	0.8296	4.1981	3.0951
Testbed 9	0.0145	6.0	0.9967	5	184	234	0.9000	1.2179	0.3965
Testbed 10	0.0410	15.0	0.6701	15	29	250	-0.4136	2.5541	0.5523
Testbed 11	0.0273	7.3	0.8319	137	82	218	0.2491	4.5938	0.9132
Testbed 12	0.0141	6.0	0.9999	11	171	250	0.9000	6.4896	1.6200
Testbed 13	0.0141	6.2	0.6700	34	122	229	0.9000	3.9462	1.0327
Testbed 14	0.0162	11.0	0.6700	48	208	246	0.2572	5.3215	1.1149
Testbed 15	0.0221	6.1	0.9814	5	72	132	0.4628	0.0070	0.0016

Appendix B. Extended Analysis with 10 Opinion-related Parameters ($O_1, \ldots, O_{10}$)

In this section, we extend our experimental design and test all ten opinion-related factors' ($O_1, \cdots, O_{10}$) impact on the disease spread. We generate 1,000 distinct tuples from the ranges shown in Table 1 using Latin hypercube sampling with the minimax correlation criterion. Again, each tuple is examined based on the 15 testbeds. All other parameter values except for the 10 opinion-related ones are fixed to theirs in the used testbed. For further analysis, we track the number of total new infections during the simulation time (Y_C) and use the log base 10 of the values as the response variable. As we found in the results with 6 parameters, the mean of the initial opinions on intervention (O_1) dominantly affects the disease spread in all 15 models again.

Table S3

Normalized permutation importance (sum to 1.0) of 10 opinion-related factors in their corresponding random forest regression models and their R^2 values. The 15 explanatory models are generated based on the results of 1,000 simulation instances each with the corresponding testbed. The result shows that, among the 10 factors, the average of the public's initial opinion values (O_1) dominates the rest in terms of the permutation importance.

Basis	R^2	Permutation Importance of Opinion Model Factors									
		O_1	O_2	O_3	O_4	O_5	O_6	O_7	O_8	O_9	O_{10}
Testbed 1	0.7888	0.7603	0.0066	0.0022	0.0067	0.0030	0.0520	0.0044	0.1592	0.0030	0.0026
Testbed 2	0.8626	0.8496	0.0003	0.0156	0.0005	0.0014	0.0775	0.0012	0.0489	0.0025	0.0024
Testbed 3	0.7987	0.7645	0.0044	0.0046	0.0025	0.0015	0.1298	0.0034	0.0656	0.0095	0.0143
Testbed 4	0.8221	0.9339	0.0006	0.0105	0.0010	0.0000	0.0405	0.0010	0.0097	0.0012	0.0015
Testbed 5	0.8974	0.8190	0.0003	0.0327	0.0012	0.0002	0.0944	0.0013	0.0495	0.0005	0.0009
Testbed 6	0.9235	0.9020	0.0004	0.0306	0.0069	0.0001	0.0493	0.0005	0.0087	0.0012	0.0003
Testbed 7	0.9256	0.8210	0.0003	0.0431	0.0006	0.0001	0.0782	0.0003	0.0540	0.0018	0.0007
Testbed 8	0.9241	0.8387	0.0003	0.0062	0.0000	0.0005	0.0567	0.0009	0.0955	0.0005	0.0006
Testbed 9	0.8204	0.7682	0.0038	0.0005	0.0050	0.0008	0.1519	0.0056	0.0499	0.0006	0.0137
Testbed 10	0.9328	0.8689	0.0003	0.0268	0.0215	0.0004	0.0563	0.0007	0.0236	0.0013	0.0001
Testbed 11	0.9401	0.9216	0.0001	0.0191	0.0015	0.0005	0.0493	0.0002	0.0070	0.0004	0.0003
Testbed 12	0.8640	0.7606	0.0036	0.0011	0.0024	0.0074	0.0527	0.0013	0.1633	0.0044	0.0033
Testbed 13	0.8171	0.8567	0.0049	0.0060	0.0013	0.0007	0.0774	0.0014	0.0478	0.0023	0.0016
Testbed 14	0.9400	0.8779	0.0003	0.0367	0.0019	0.0000	0.0648	0.0018	0.0158	0.0006	0.0002
Testbed 15	0.8697	0.7710	0.0039	0.0193	-0.0001	0.0010	0.0765	0.0021	0.1206	0.0040	0.0019

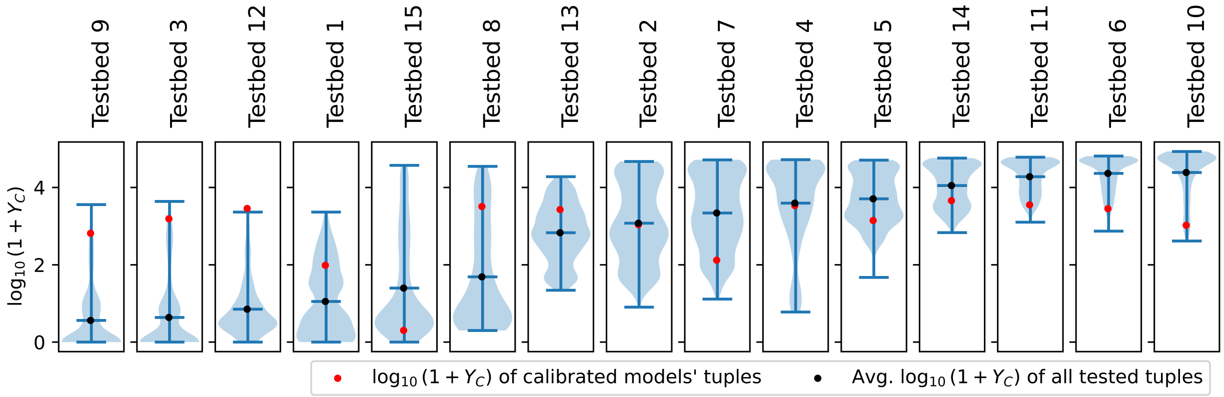

Fig. S1. Violin plots of the log-scaled epidemic size (Y_C) distributions of 1000 opinion-related parameter tuples in baseline models fitted to the data for 15 countries. Plots are sorted by the mean of the log-scaled epidemic size. Epidemic sizes are measured in the number of total new infections during the simulation time in a virtual social system with a 50,000 population size. The red dots in the figure represent the response values (Y_C) of the models fitted to the corresponding data. Plots show that the consequence could be much better or much worse depending on the given conditions of opinion dynamics.

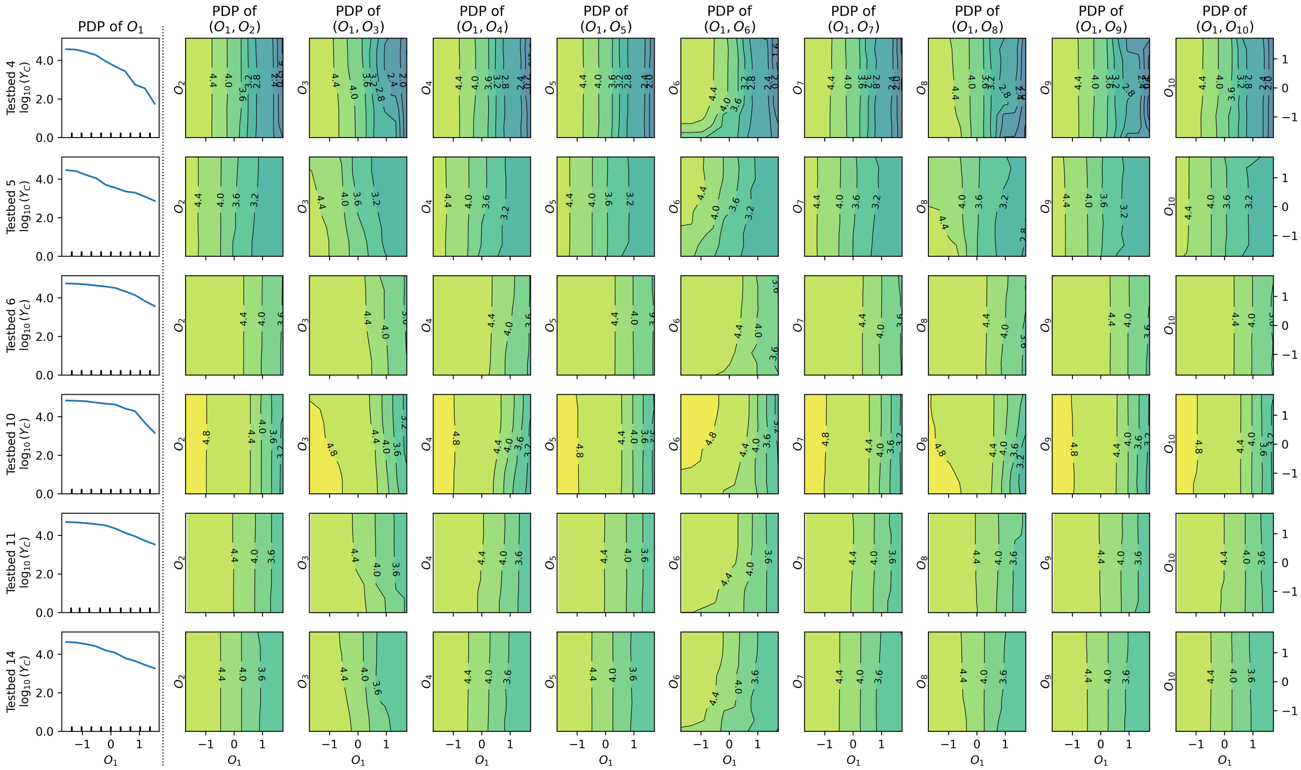

Fig. S2. One one-way (O_1) and nine two-way partial dependence plots of the regression model for the cases in Group 1: Results based on Testbeds 4, 5, 6, 10, 11, and 14.

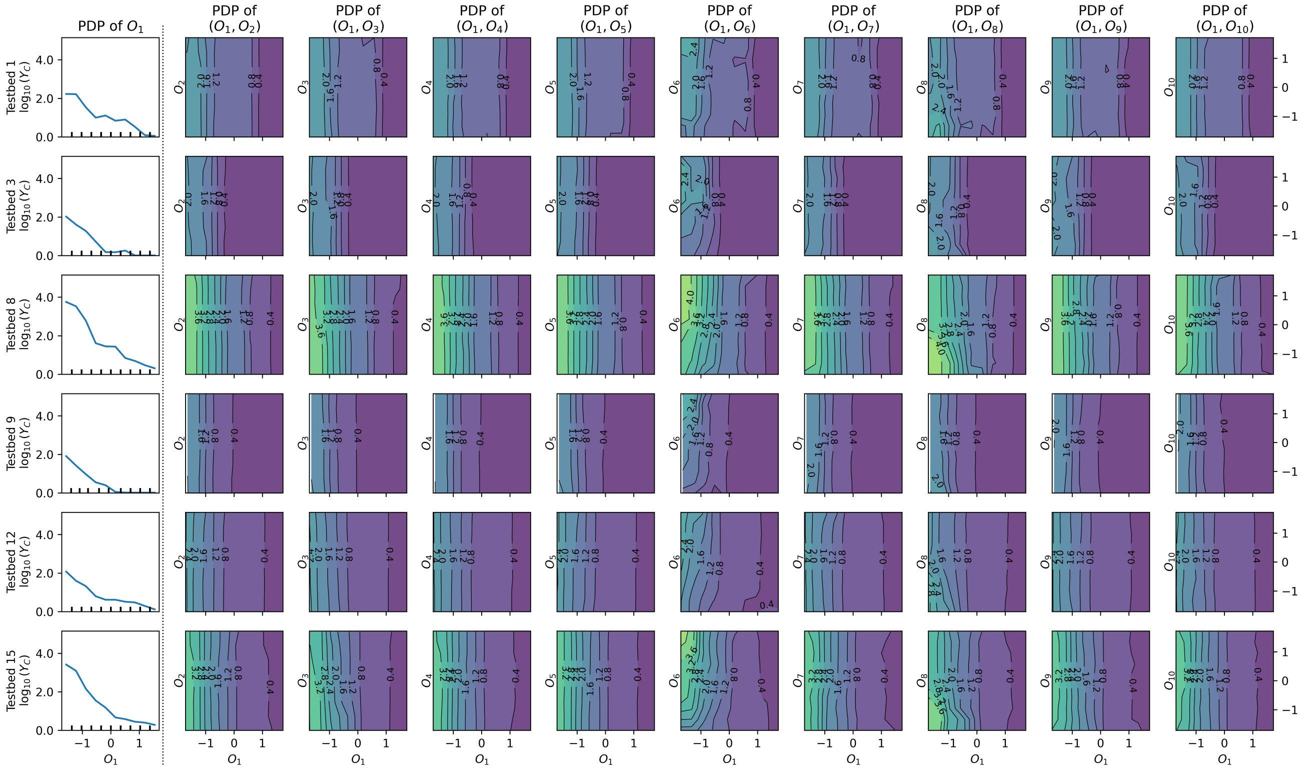

Fig. S3. One one-way (O_1) and nine two-way partial dependence plots of the regression model for the cases in Group 2: Results based on Testbeds 1, 3, 8, 9, 12, and 15.

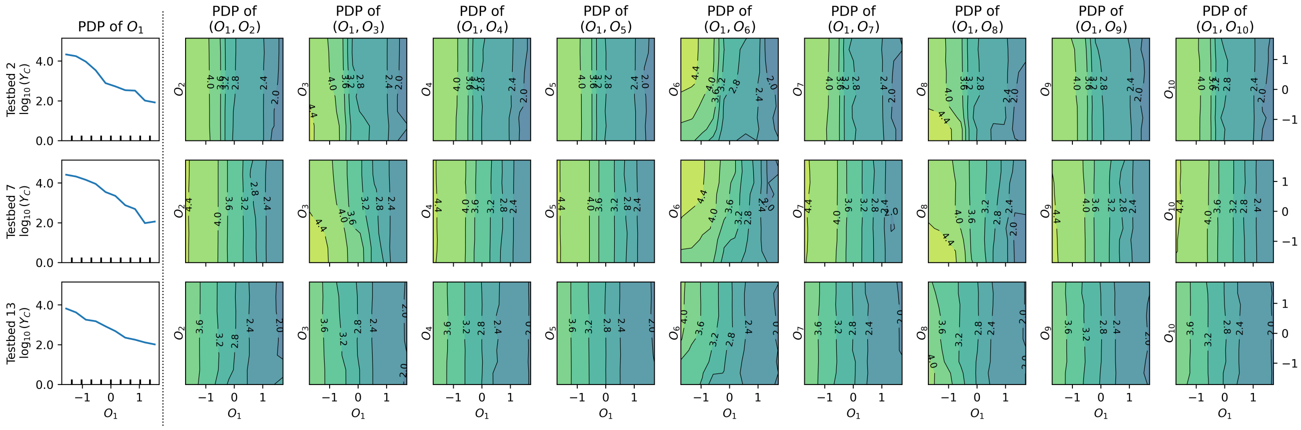

Fig. S4. One one-way (O_1) and nine two-way partial dependence plots of the regression model for the cases in Group 3: Results based on Testbeds 2, 7, and 13.
